# Two-week step-reduction has limited negative effects on physical function and metabolic health in older adults

**DOI:** 10.1007/s00421-024-05426-1

**Published:** 2024-02-21

**Authors:** Simon Walker, Ulla-Maria Sahinaho, Sakari Vekki, Mari Sulonen, Jari A. Laukkanen, Sarianna Sipilä, Heikki Peltonen, Eija Laakkonen, Maarit Lehti

**Affiliations:** 1 Faculty of Sport and Health Sciences, University of Jyväskylä, Room VIV225, 40014-FI Jyväskylä, Finland; 2 NeuroMuscular Research Center, University of Jyväskylä, Jyväskylä, Finland; 3 Institute of Clinical Medicine, Department of Medicine, University of Eastern Finland, Kuopio, Finland; 4 Department of Medicine, Wellbeing Services County of Central Finland, Jyväskylä, Finland; 5 Gerontology Research Center, University of Jyväskylä, Jyväskylä, Finland; 6 JAMK University of Applied Science, The School of Business, Sport Business, Jyväskylä, Finland

**Keywords:** Physical activity, Inactivity, HDL, Exercise, Walking economy, Strength

## Abstract

**Purpose:**

This study determined the effects of a 2-week step-reduction period followed by 4-week exercise rehabilitation on physical function, body composition, and metabolic health in 70–80-year-olds asymptomatic for injury/illness.

**Methods:**

A parallel-group randomized controlled trial (ENDURE-study, NCT04997447) was used, where 66 older adults (79% female) were randomized to either intervention or control group. The intervention group reduced daily steps to < 2000, monitored by accelerometer, for two weeks (Period I) and then step-reduction requirement was removed with an additional exercise rehabilitation 4 times per week for 4 weeks (Period II). The control group continued their habitual physical activity throughout with no additional exercise intervention. Laboratory tests were performed at baseline, after Period I and Period II. The primary outcome measure was leg lean mass (LLM). Secondary outcomes included total lean and fat mass, blood glucose and insulin concentration, LDL cholesterol and HDL cholesterol concentration, maximal isometric leg press force (MVC), and chair rise and stair climb performance.

**Results:**

LLM remained unchanged in both groups and no changes occurred in physical function nor body composition in the intervention group in Period I. HDL cholesterol concentration reduced after Period I (from 1.62 ± 0.37 to 1.55 ± 0.36 mmol·L^−1^, *P* = 0.017) and returned to baseline after Period II (1.66 ± 0.38 mmol·L^−1^) in the intervention group (Time × Group interaction: *P* = 0.065). MVC improved after Period II only (Time × Group interaction: *P* = 0.009, Δ% = 15%, *P* < 0.001).

**Conclusion:**

Short-term step-reduction in healthy older adults may not be as detrimental to health or physical function as currently thought.

## Introduction

In older age (i.e., > 60 years), cross-sectional studies have reported that differences in maximal force production and muscle mass are in the magnitude of ~ 10–30% and ~ 5–10% per decade, respectively (Frontera et al. 1991; Häkkinen and Häkkinen 1991; Lindle et al. 1997). Such observations have been supported by small-sample longitudinal studies, as losses of ~ 20% in maximal force production and ~ 10% reductions in muscle mass occurred over a 10-year period (Frontera et al. 2000). Since low levels of maximum force production and muscle mass accompany progression of disability (Rantanen et al. 1999), this is of concern for progressively aging societies.

While the aforementioned declines appear to be gradual, linear processes, it has been proposed that short periods of physical inactivity/muscle disuse occur at a higher frequency in older (than younger) adults, and it is these periods that lead to the reported decade-long losses (Bell et al. 2016). A short-term period of physical inactivity could occur due to illness, injury, inclement weather (e.g., cold and icy conditions), or indeed during the recent COVID-19 pandemic when > 70-year-olds were quarantined in some countries. Thus, knowledge regarding the health-related consequences of short-term physical inactivity in older adults, and their ability to overcome the deleterious effects of these periods, is needed.

Complete bed rest or immobilization is an extreme form of physical inactivity, and this has been shown to reduce maximum force production and muscle mass, as well as increase glucose intolerance/insulin resistance within days (Reidy et al. 2020; Suetta et al. 2012). However, perhaps a more ecologically valid model of short-term reduced physical activity in older adults is a step-reduction approach. Using this model, initial studies in young adults showed that lowering daily steps < 1500 led to abnormal responses to an oral glucose tolerance test (Olsen et al. 2008), reduced cardiorespiratory fitness (Krogh-Madsen et al. 2010), and increased intra-abdominal fat and reduced leg lean mass (Krogh-Madsen et al. 2010; Olsen et al. 2008) over a 2–3-week period.

Since then, both supportive and conflictive findings have been observed in older adults, for example, reducing daily steps to ~ 1500 led to 1.5–4% loss in leg lean mass (Breen et al. 2013; Devries et al. 2015) but reducing daily steps to < 1000 over a 2-week period did not lead to significant lean mass reductions in one study (McGlory et al. 2018). Despite this conflict, consistent findings related to reduced glucose regulation have been observed over 2-week step-reduction periods (Breen et al. 2013; McGlory et al. 2018; Saoi et al. 2019), which may even precede body composition changes (Knudsen et al. 2012). The remaining unexplored aspect of the step-reduction literature in older adults is a comprehensive examination of potential loss in physical function.

Further, simply allowing participants to return to their normal habitual physical activity level does not fully reverse the effects of step-reduction (McGlory et al. 2018). Despite few studies including an exercise intervention during or after the step-reduction period to offset the expected declines, these have led to different levels of success (Devries et al. 2015; Saoi et al. 2019). Therefore, examination of exercise interventions to reverse short-term physical inactivity is also warranted.

The purpose of the present study was to determine (1) the effects of a 2-week step-reduction period on physical function, body composition, and metabolic health in asymptomatic 70–80-year-olds and (2) the effects of a 4-week exercise rehabilitation period on the examined variables. Based on previous evidence (Breen et al. 2013; Devries et al. 2015; McGlory et al. 2018; Saoi et al. 2019), it was hypothesized that two weeks of step-reduction (< 2000 steps per day) would lead to reductions in leg lean mass, lower-limb force production capacity, increases in fat mass, and altered markers of metabolic health.

## Methods

### Study design and setting

This study was a two-arm, parallel-group randomized controlled trial (ENDURE-study, NCT04997447). This study was conducted according to the Declaration of Helsinki and was approved by the Ethics Committee of the Central Finland Health Care District (3U/2021). Measurements were performed at baseline, after two weeks (Period I), and then after a further four weeks (Period II). All volunteers (*n* = 78) provided written informed consent prior to lab-based health examinations. The primary outcome measure of ENDURE was leg lean mass (LLM).

### Recruitment

Recruitment occurred via local advertisements and social media in the city of Jyväskylä, Finland. Potential participants were assessed for initial inclusion criteria by telephone interview, checking their current physical activity and fitness, musculoskeletal disorders, medical history, current/permanent conditions, medications, and risk factors of cardiovascular diseases (e.g., smoking, high blood pressure or cholesterol, family history of cardiovascular events, and severe obesity). All information of this study was then sent to suitable individuals, including possible risks and harms as well as data confidentiality management.

**Fig. 1:**
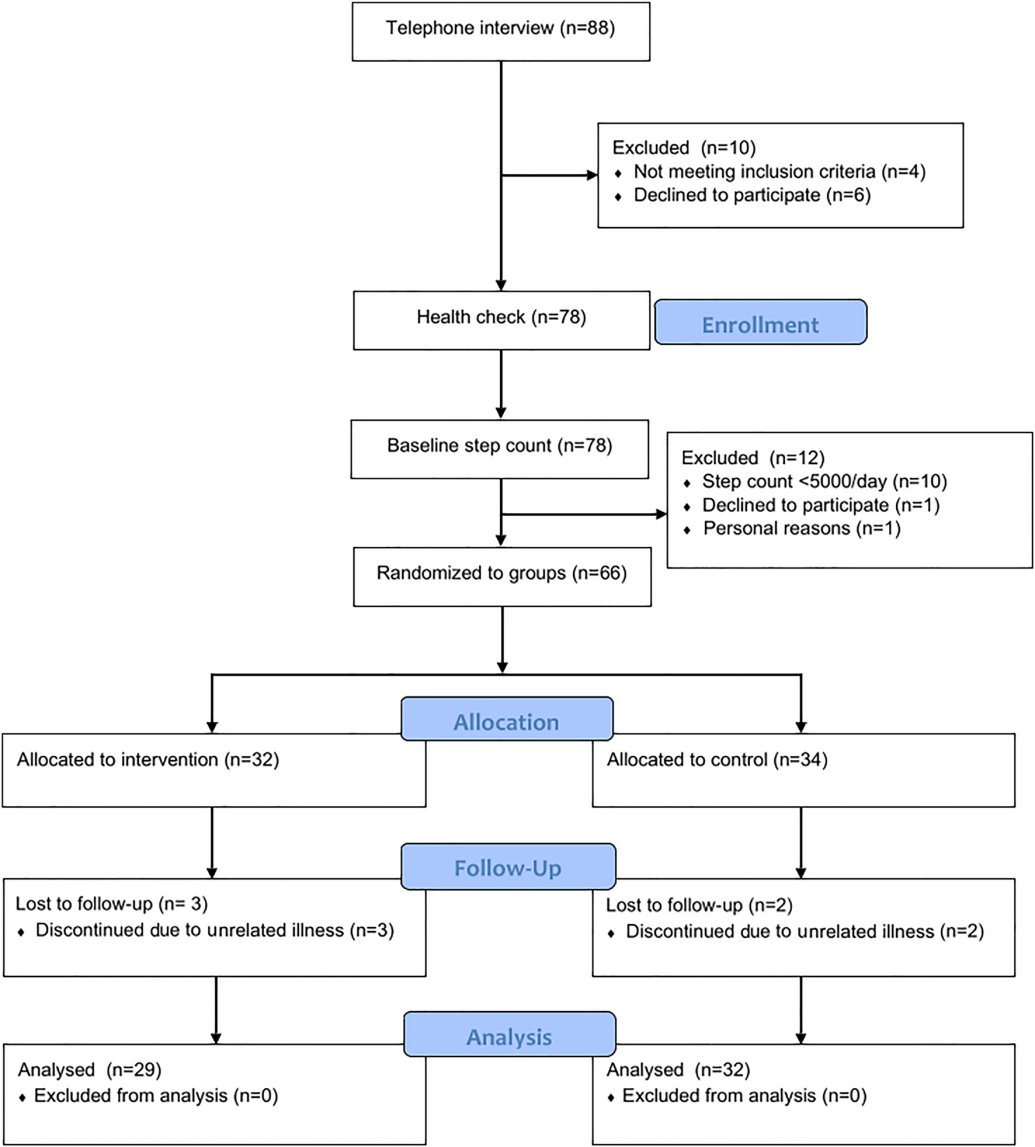
Study flowchart from the point of initial screening of the volunteers after expression of interest to participate

Prior to study initiation and acceptance to the study as a participant, all interested volunteers (*n* = 78) underwent a health examination including resting electrocardiography, blood pressure, body mass index, and Mini Mental State Examination (Fig. 1). After the physician’s approval, volunteers were screened over a 5-day period where normal physical activity was tracked with activity diary. Daily step count was measured with a hip-worn accelerometer (UKK RM42, UKK Terveyspalvelut Oy, Tampere, Finland). The accelerometer was worn during waking hours, excluding bathing. Accelerometer data were converted into step count using Actigraph Actilife software (Actigraph LLC, Florida, USA). Volunteers were blind to the purpose of the step count, which was to discount persons who habitually walked < 5000 steps per day. After baseline step count, the remaining suitable volunteers (*n* = 66) were accepted to this study and continued to baseline laboratory tests.

### Inclusion and exclusion criteria

Eligible participants were males and females fulfilling the following inclusion criteria: (1) aged 70–80 years, (2) community-dwelling, (3) able to walk 500 m without assistance or use of walking aid and regularly walking > 5000 steps per day, (4) Mini Mental State Examination score > 23, (5) body mass index 20–35 kg·m^−2^, (6) no serious, symptomatic cardiovascular or musculoskeletal disease, (7) no risk factors for deep-vein thrombosis (e.g., blood clotting disorder, severe obesity, bowel diseases), (8) non-smoker, and (9) provision of informed consent.

Exclusion criteria were as follows: (1) underlying diseases likely to limit lifespan and/or intervention safety, (2) contraindication for physical exercise or physical tests identified during physician’s examination, (3) unwilling/unable to track daily step counts using accelerometer, (4) excessive and regular use of alcohol (more than 7 units per week for women and 14 for men; 1 unit is equivalent to 10 ml of pure alcohol), (5) difficulty in communication due to severe vision or hearing problems, and (6) unwilling to provide consent or accept randomization into either study group.

### Randomization

Sixty-six participants were randomly divided to an intervention or control group using a computer-generated random number sequence. Following drop-outs (*n* = 5), the final study groups entered into further analyses consisted of 29 participants (males, *n* = 6; females, *n* = 23) in the intervention group and 32 participants (males, *n* = 7; females, *n* = 25) in the control group in total (Fig. 1). All participants were advised to continue their normal dietary intake for the duration of the study.

### Sample size calculations

Sample size estimations are based on the primary outcome measure (leg lean mass) and calculated based on effect sizes obtained from Breen et al. (2013) for (3)Time × (2)Group interactions with an alpha of 0.05 and power level of 0.95 (G*Power software, Dusseldorf, Germany). Within-subject correlations for the repeated measures were assumed to be *r* = 0.8. Consequently, a sample of forty-eight (24 per group) was needed, while drop-out and potential non-compliance to step-reduction was expected to be 20%.

### Interventions

Step-reduction protocol: During the 2-week step-reduction period, the intervention group was instructed to decrease daily steps below 2000 while the control group continued their normal habitual physical activity behavior. Daily steps were tracked by accelerometer (UKK RM 42) and step count was calculated with Actigraph Actilife software (Actigraph LLC, Florida, USA). Simultaneously, to provide visual feedback of daily steps and record anomalies, the intervention group participants were also provided pedometers (Omron Walking Style One, HJ-152R-S) and an activity diary, e.g., if they removed the accelerometers during the day.

During the step-reduction period, the intervention group were phoned once per week to determine whether step-reduction led to any health consequences requiring medical attention. Participants were also instructed to mobilize the ankle joint while sitting (i.e., venous pump exercises) throughout the day.

Exercise rehabilitation protocol: The requirement to restrict daily steps to 2000 was removed for the intervention group and they could return to their habitual physical activity behavior. In addition, the intervention group performed whole-body resistance training twice a week, as well as cycle ergometer-based endurance training twice a week (see supplementary material for exact intervention details). Exercise sessions took place in the University’s facilities. Endurance training was performed on Precor Teambike (Precor Inc., Seattle, USA) and began by a 5-min warm-up at 40–50% of maximum theoretical age-based heart rate (HRmax) according to the equation by Tanaka et al. (2001). Participants continued for 25 min at steady pace and resistance keeping to 50–60% HRmax. This was followed by 5 min (pyramid) intervals at 60–70% HRmax, 75–95% HRmax, and then 60–70% HRmax. The cool-down period was 10 min at 50–60% HRmax, making a total exercise duration of 55 min. Heart rate was tracked in real time using Polar V800 monitor connected to H7 sensor (Polar Electro Oy, Kempele, Finland).

Resistance training consisted of 9 exercises encompassing all major muscle groups. Lower limbs were always trained first by leg press, knee extension, knee flexion, and straight-legged calf raise. Thereafter, chest press, lat pulldown, abdominal bench, and cervical extension were performed, with biceps curl being trained on one day while triceps pushdown being trained on the other day. The program consisted of 2–3 sets of 12–14 repetitions with 2-min inter-set rest. Loads were increased throughout the 4-week period whenever 14 repetitions were accomplished in the last set. All exercise sessions were supervised by members of the research team.

### Primary outcome

LLM was the primary outcome measure as assessed by dual-energy X-ray absorptiometry (DXA) after an overnight (~ 12-h) fast. The coefficient of variation for repeated measures for this test has been reported as 2.2% for fat percentage and 1% for lean tissue mass (Sillanpää et al. 2014).

### Secondary outcomes

Body composition: Participants underwent full-body DXA scanning in minimal clothing (LUNAR Prodigy Advance with Encore software version 9.3, GE medical systems, United States). The legs were separated by a polystyrene block and fixated, while the arms were separated from the trunk (Walker et al. 2017). Total body fat mass (TFM), total body lean mass (TLM), and LLM were determined using software-generated analyses. The DXA measurements were performed in a fasted state in the morning (07:00–09:30 h).

Functional capacity: Maximal bilateral voluntary contraction (MVC) of the leg extensors was assessed using a custom-built electromechanical (leg press) dynamometer. Participants were positioned so that the knee angle was 107 degrees, and they were instructed to push against the foot plate “as hard and as fast as possible” while under loud encouragement. Force signals were sampled at 2000 Hz (Signal 4.14, CED, UK) and filtered by a 10 Hz low-pass (Butterworth 4th order) filter. Analysis was performed using a customized script to identify the instantaneous peak force. Reliability for MVC using this device in our laboratory in older adults has been reported as intra-class correlation coefficient = 0.922, coefficient of variation % = 4.2% (Walker & Häkkinen 2014).

Functional capacity was measured by a short physical performance test battery (SPPB). The battery included standing balance, habitual walking speed, and lower extremity strength (Guralnik et al. 1994). Standing balance included stances of feet together, semi-tandem, and full tandem for a maximum of 10 s. In the 5 sit-to-stand test (Chair), participants were required to sit and stand five times as rapidly as possible. Each participant began seated with their back against an armless chair and arms crossed over their chest. The timer was initiated upon moving to stand and stopped when the participant returned to the seat following the fifth stand. In the 4 m walking test, participants walked 4 m forward with the time recorded for both normal walking speed (Norm4). Additionally, participants performed a maximum walking speed (Max4) test and a 10-stair climb test (Stair), where the participants ascended 10 steps as quickly and safely as possible as previously reported (Turpela et al. 2017). In Stair, the participants carried one bag of 5 kg (females) or 10 kg (males) in each hand and the climb time was recorded by custom-built photocells.

Walking economy was measured on a treadmill (OJK-1, Telineyhtymä, Kotka, Finland). The test began with quiet standing for 2 min, then continued by walking at a speed of 3 km/h for 4 min, and then for 4 min at 5 km/h (Delabastita et al. 2021). Respiratory gases were collected breath-by-breath using a metabolic cart (Vyntus CPX, Vyaire, Hoechberg, Germany) and data averaged over the final 60 s of each stage were taken forward for further analyses. Carbohydrate and fat oxidation rates were estimated from respiratory gases using Frayn’s (1983) equations with the assumption that urinary nitrogen excretion was negligible (i.e., amino acids were not used in synthesizing glucose):

Carbohydrate oxidation rate (g·min^−1^) = 4.55 × VCO_2_ (l min^−1^)–3.21 × VO_2_ (l min^−1^),

Fat oxidation rate (g·min^−1^) = 1.67 × VO_2_ (l min^−1^)–1.67 × VCO_2_ (l min^−1^).

A heart rate monitor (Polar H10, Polar Electro Oy, Kempele, Finland) was placed at the sternum and synchronized with the metabolic cart.

Blood sampling procedures and blood-based metabolic health markers: Blood samples were collected in the morning after a 12-h overnight fast. Venous blood samples were collected into 3 ml tubes for whole blood analyses (Vacuette K3EDTA, Greiner Bio-One GmbH, Kremsmünster, Austria). Hematocrit was immediately analyzed from the whole blood with cell counter (Sysmex, models KX-21N and XP-300, TOA Medical Electronics Co., Ltd., Kobe, Japan). Venous blood was collected into 6 ml tubes containing clot activator for serum analyses (Vacuette, Greiner Bio-One GmbH, Kremsmünster, Austria). The samples stood at room temperature for 15 min before being centrifuged for 15 min at 3600 rpm (2245 rcf, Heraeus Megafuge 1.0 R, Heraeus Holding GmbH, Hanau, Germany). Serum samples were analyzed for glucose (Glucose (HK), Cat. No. 981779, Thermo Scientific Inc., Vantaa, Finland), insulin (IMMULITE 2000 Insulin, Cat. No. L2KIN6, Siemens Healthcare Diagnostics Ltd., Llanberis, United Kingdom), uric acid (Uric acid (AOX), Thermo Fisher Scientific Inc., Vantaa, Finland), and lipid profile including total cholesterol, HDL cholesterol (HDL-C), LDL cholesterol (LDL-C), and triglycerides (Cholesterol, Cat. No. 981813; HDL-C Plus, Cat. No. 981823; LDL-C, Cat. No. 981956; Triglycerides, Cat. No. 981786, Thermo Fisher Scientific Inc., Vantaa, Finland). All serum analyses were performed with Indiko Analyzer (Indiko Plus, Thermo Fisher Scientific Inc., Vantaa, Finland).

### Adverse effects

Participants reported new symptoms, injuries, and/or diseases to the study coordinator throughout the 6-week period. Reported adverse effects were logged in an electronic record sheet.

### Statistical analyses

Statistical analyses were performed using SPSS (version 28, IBM SPSS Statistics, USA) software and alpha was set to 0.05. Analyses were performed using the intention-to-treat principle with the exception of the 5 drop-outs that occurred during this study. Thus, the final *n* for the intervention group was 29 and the *n* for the control group was 32. These sample sizes were included to the statistical tests unless otherwise stated. Data are reported as mean ± standard deviation unless otherwise stated.

Data normality was examined through the Shapiro–Wilk test and homogeneity of variance by Levene’s test. Most variables were not normally distributed; therefore, Log_10_ transformation was performed prior to parametric statistical tests. Baseline characteristics were examined by independent t-test for possible between-group differences. Where sphericity was not assumed, Greenhouse–Geisser adjustments were applied to the degrees of freedom and noted when reporting the F-value. Repeated measures analysis of variance (ANOVA) using a (3)Time × (2)Group design was used to assess the study’s dependent variables. When a significant F-value was observed, Bonferroni post hoc tests were applied to determine the locality of the difference. Nevertheless, due to low compliance (12/29, 41%) with the < 2000 steps per day requirement, secondary analyses were performed comparing these “compliers” (*n* = 12) to the “non-compliers” (*n* = 17) and control (*n* = 32) in a (3)Time × (3)Group design.

## Results

**Fig. 2:**
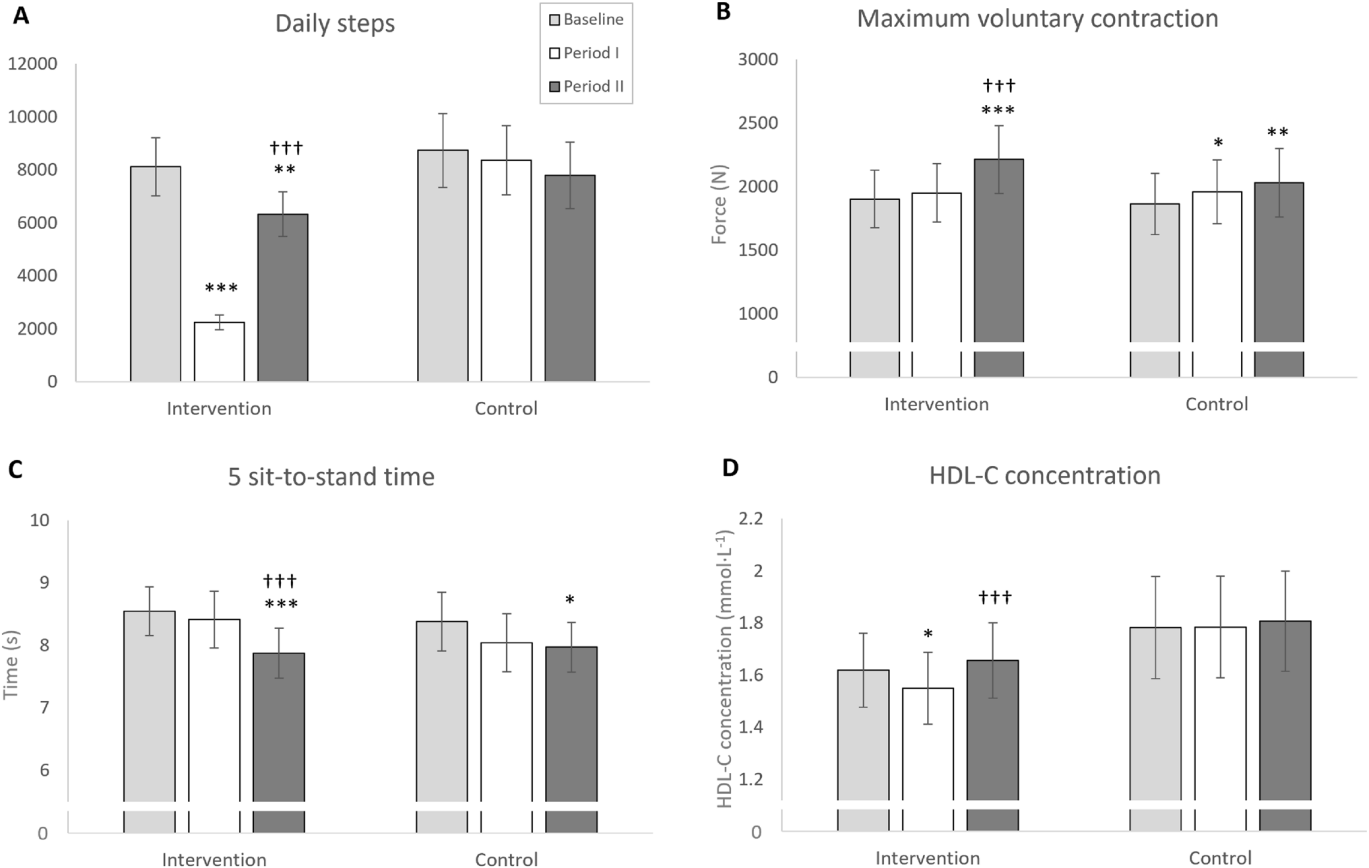
Mean ± 95% confidence intervals for daily steps (**A**), maximal bilateral isometric leg press voluntary contraction force (**B**), 5 sit-to-stand time (**C**), and high-density lipoprotein cholesterol concentration (**D**) in the intervention and control groups. *P < 0.05, **P < 0.01, ***P < 0.001 compared to baseline. †††*P* < 0.001 compared to Period I

At baseline, the participant characteristics of the intervention and control groups did not differ (Table 1). Daily steps demonstrated significant main effects for Time (F_(2, 118)_ = 87.109, *P* < 0.001), Group (F_(1, 59)_ = 42.953, *P* < 0.001), and a Time × Group interaction (F_(2, 118)_ = 86.461, *P* < 0.001). The intervention group reduced daily steps from baseline (8121 ± 2872 steps) to Period I (2242 ± 727 steps, Δ–68 ± 16%, *P* < 0.001) and they remained lowered during Period II (6328 ± 2214 steps, Δ–17 ± 30%, *P* = 0.003). No change was observed in the Control group (Baseline: 8732 ± 3861, Period I: 8369 ± 3627, Period II: 7790 ± 3483) (Fig. 2A).

**Table 1:** Baseline characteristics displayed as mean ± SD

	Intervention (*n* = 29)	Control (*n* = 32)	Between-group *P*-value
Age (years)	72.9 ± 2.8	72.2 ± 2.3	0.327
Women, no./%	23/79%	25/78%	
Height (m)	1.66 ± 7.88	1.64 ± 8.88	0.369
Body mass (kg)	72.3 ± 12.0	70.4 ± 12.4	0.546
BMI (kg·m^−2^)	26.2 ± 3.4	26.1 ± 3.5	0.940
Lean leg mass (kg)	14.0 ± 2.4	13.4 ± 3.7	0.197
Blood pressure sys/dia (mmHg)	137 ± 9/79 ± 7	134 ± 10/ 77 ± 8	0.192/0.358
SPPB points	12.0 ± 0.2	12.0 ± 0.0	0.305
Daily steps	8121 ± 2872	8732 ± 3861	0.632
Medications, no./%			
Statins	4/14%	4/13%	
Antihyperglycemic drugs	1/3%	2/6%	
Beta-blockers	1/3%	3/9%	
Calcium channel blockers	3/10%	1/3%	
Angiotensin II receptor blockers	4/14%	6/19%	
Anticoagulants	1/3%	3/9%	
Thyroid replacement therapy	6/21%	4/13%	
Estrogens (HRT)	2/7%	2/6%	
Estrogen-receptor modulators (SERMs)	0/0%	1/3%	
Prostatic hyperplasia inhibitors	2/7%	1/3%	
Corticosteroids	1/3%	0/0%	
Anti-rheumatic drugs	1/3%	1/3%	
Anti-resorptive drugs (ARDs)	0/0%	2/6%	
Calcium	0/0%	3/9%	
Bronchodilators	5/17%	3/9%	

*BMI* body mass index, *sys/dia* systolic/diastolic, *SPPB* short physical performance battery, *HRT* hormone replacement therapy

### Outcomes

There were no significant main effects observed for the primary outcome measure (LLM) (Time: F_(1.7, 97.7)_ = 0.370, *P* = 0.652, Time × Group: F_(1.7, 97.7)_ = 0.031, *P* = 0.949, Group: F_(1, 59)_ = 1.125, *P* = 0.293, Table 2). Conversely, a main effect for Time (F_(2, 118_ = 30.15, *P* < 0.001) and a Time × Group interaction (F_(2, 118_ = 4.96, *P* = 0.009) was observed in MVC. Here, the control group improved from baseline to Period I (*P* = 0.040) and this improvement was maintained after Period II (*P* = 0.002, Fig. 2B). The intervention showed no change from baseline to Period I (*P* = 0.499), but then increased MVC from Period I to Period II (*P* < 0.001), such that there was a significant difference from baseline also (*P* < 0.001, Table 2).

**Table 2:** Body composition and physical function at baseline and following the step-reduction (Period I) and exercise rehabilitation (Period II) periods. Data displayed as mean ± SD

		Intervention	Control	Time *P*-value	Group *P*-value	Time × Group interaction *P*-value
LLM (kg)	Baseline	14.0 ± 2.4	13.4 ± 3.7	0.652	0.293	0.949
	Period I	14.1 ± 2.4	13.5 ± 3.7			
	Period II	14.0 ± 2.3	13.4 ± 3.7			
TLM (kg)	Baseline	44.2 ± 7.0	42.4 ± 9.8	0.241	0.264	0.573
	Period I	44.3 ± 7.0	42.4 ± 9.7			
	Period II	44.5 ± 7.2	42.5 ± 9.9			
TFM (kg)	Baseline	24.4 ± 9.1	23.5 ± 7.2	0.045	0.264	0.573
	Period I	24.5 ± 8.9	23.6 ± 7.4			
	Period II	24.0 ± 9.0†	23.5 ± 7.4			
MVC (N)	Baseline	1901 ± 595	1864 ± 667	< 0.001	0.542	0.009
	Period I	1949 ± 604	1959 ± 700*			
	Period II	2213 ± 703*†	2030 ± 743*			
F100 (N)	Baseline	336 ± 248	281 ± 209	< 0.001	0.303	0.451
	Period I	364 ± 220	350 ± 237*			
	Period II	414 ± 226*	355 ± 207*			
Norm4 (s)	Baseline	2.75 ± 0.39	2.78 ± 0.37	< 0.001	0.567	0.317
	Period I	2.65 ± 0.28	2.65 ± 0.39			
	Period II	2.48 ± 0.35*	2.59 ± 0.35*			
Max4 (s)	Baseline	1.82 ± 0.19	1.81 ± 0.30	0.026	0.954	0.366
	Period I	1.79 ± 0.17	1.79 ± 0.23			
	Period II	1.74 ± 0.16*	1.77 ± 0.24			
Chair (s)	Baseline	8.55 ± 1.01	8.38 ± 1.30	< 0.001	0.557	0.059
	Period I	8.41 ± 1.18	8.04 ± 1.27			
	Period II	7.87 ± 1.06*†	7.97 ± 1.09*			
Stair (s)	Baseline	3.51 ± 0.47	3.56 ± 0.78	< 0.001	0.938	0.297
	Period I	3.42 ± 0.41	3.42 ± 0.65			
	Period II	3.32 ± 0.41*	3.43 ± 0.68			

*MVC* maximal voluntary contraction, *F100* force production over the initial 100 ms of isometric contraction, *Norm4* normal-speed walking over 4 m, *Max4* maximum-speed walking over 4 m, *Chair* 5 sit-to-stand time, *Stair* 10-step stair climb time, *LLM* lean mass of the legs, *TLM* total lean mass, *TFM* total fat mass

*Statistically significant difference compared to baseline, *P* < 0.05

†Statistically significant difference compared to Period I, *P* < 0.05

Main effects for Time were also observed in F100 (F_(1.8, 106.7)_ = 18.5, *P* < 0.001), Norm4 (F_(2, 118)_ = 14.2, *P* < 0.001), Max4 (F_(1.8, 106.1)_ = 3.96, *P* = 0.026), Chair (F_(1.8, 104.2)_ = 15.29, *P* < 0.001), and Stair (F_(2, 118)_ = 8.37, *P* < 0.001). The intervention group improved functional performance in all these variables from baseline to Period II, and also from Period I to Period II in Chair (*P* < 0.001, Fig. 2C). The control improved from baseline to Period II in F100, Norm4, and Chair (Table 2). Finally, statistically significant, but small-magnitude, changes in fat mass were observed from Period I to Period II in total fat mass in the intervention group (*P* = 0.009, Table 2).

When walking at 3 km·h^−1^, significant Time × Group interactions were observed in HR (F_(1.7, 101.7)_ = 4.63, *P* = 0.016) and RER (F_(2, 118)_ = 3.61, *P* 0.031). In HR, the control group reduced from baseline to Period I (*P* = 0.026), whereas the intervention group reduced from Period I to Period II (*P* < 0.001). The intervention group demonstrated lowered RER from Period I to Period II (*P* 0.045). The oxygen consumption of walking at 3 km·h^−1^, i.e., walking economy, was reduced in both groups from Period I to Period II (intervention: *P* = 0.005, control: *P* = 0.009), leading to a difference compared to baseline also (intervention: *P* < 0.001, control: *P* = 0.024, Table 3).

**Table 3:** Physiological responses to treadmill walking at 3 km·h^−1^ at baseline and following the step-reduction (Period I) and exercise rehabilitation (Period II) periods. Data displayed as mean ± SD

		Intervention	Control	Time *P*-value	Group *P*-value	Time × Group interaction *P*-value
HR (bpm)	Baseline	106 ± 21	104 ± 21	< 0.001	0.798	0.016
	Period I	103 ± 20	98 ± 18*			
	Period II	96 ± 18*†	99 ± 18			
VO_2_ (ml·min^−1^)	Baseline	865 ± 200	880 ± 206	< 0.001	0.676	0.877
	Period I	829 ± 147	849 ± 151*			
	Period II	787 ± 146*†	810 ± 153*†			
RER (VCO_2_/O_2_)	Baseline	0.826 ± 0.052	0.814 ± 0.045	0.752	0.197	0.031
	Period I	0.839 ± 0.048	0.809 ± 0.048			
	Period II	0.813 ± 0.036†	0.804 ± 0.054			
EE (kJ)	Baseline	4.18 ± 0.97	4.22 ± 0.98	< 0.001	0.727	0.744
	Period I	4.02 ± 0.71	4.08 ± 0.73			
	Period II	3.97 ± 0.71*†	3.91 ± 0.74*†			
CHOox (g·min^−1^)	Baseline	0.475 ± 0.255	0.431 ± 0.198	0.004	0.472	0.328
	Period I	0.497 ± 0.183	0.398 ± 0.188			
	Period II	0.386 ± 0.149	0.435 ± 0.208*†			
FATox (g·min^−1^)	Baseline	0.251 ± 0.098	0.274 ± 0.095	0.701	0.232	0.092
	Period I	0.226 ± 0.080	0.270 ± 0.081			
	Period II	0.245 ± 0.063	0.238 ± 0.082			

*HR* heart rate, *VO*_*2*_ oxygen consumption, *RER* respiratory exchange ratio, *EE* energy expenditure, *CHOox* carbohydrate oxidation, *FATox* fat oxidation

*Statistically significant difference compared to baseline, *P* < 0.05

†Statistically significant difference compared to Period I, *P* < 0.05

A similar pattern of changes in HR, oxygen consumption, and energy expenditure were observed when walking at 5 km·h^−1^, although the only significant Time × Group interaction was in FATox (F_(1.76, 95.2)_ = 3.71, *P* = 0.033, Table 3). Here, increased fat oxygenation from Period I to Period II was significant in the intervention group (*P* = 0.011).

In blood-based markers of metabolic health, HDL-C demonstrated a significant main effect for Time (F_(1.78, 105.2_ = 5.80, *P* = 0.006), but not Time × Group interaction (F_(1.78, 105.2_ = 2.91, *P* = 0.065, Table 4). Here, the intervention group showed reduced HDL-C concentration from baseline to Period I (*P* = 0.017), which then increased from Period I to Period II (*P* < 0.001, Fig. 2D). In LDL-C, a significant main effect for Time was observed (F_(2, 118)_ = 38.37, *P* < 0.001). Here, both groups increased LDL-C concentration from baseline to Period I (intervention: *P* < 0.001, control: *P* = 0.036) and then the control group further increased from Period I to Period II (*P* = 0.001) (Table 5).

**Table 4:** Physiological responses to treadmill walking at 5 km·h^−1^ at baseline and following the step-reduction (Period I) and exercise rehabilitation (Period II) periods. Data displayed as mean ± SD

HR (bpm)	Baseline	124 ± 19	118 ± 22	< 0.001	0.257	0.542
	Period I	120 ± 20	113 ± 21*			
	Period II	112 ± 18*†	109 ± 21*			
VO_2_ (ml·min^−1^)	Baseline	1194 ± 226	1180 ± 245	< 0.001	0.805	0.779
	Period I	1161 ± 193	1165 ± 210			
	Period II	1113 ± 213*†	1092 ± 186*†			
RER (VCO_2_/O_2_)	Baseline	0.878 ± 0.074	0.860 ± 0.066	0.059	0.244	0.383
	Period I	0.880 ± 0.057	0.856 ± 0.086			
	Period II	0.857 ± 0.059	0.850 ± 0.054			
EE (kJ)	Baseline	5.86 ± 1.20	5.76 ± 1.23	< 0.001	0.753	0.844
	Period I	5.69 ± 0.98	5.68 ± 1.09			
	Period II	5.42 ± 1.10*†	5.31 ± 0.93*†			
CHOox (g·min^−1^)	Baseline	0.974 ± 0.645	0.848 ± 0.469	0.141	0.733	0.584
	Period I	0.927 ± 0.421	0.823 ± 0.598			
	Period II	0.786 ± 0.460	0.730 ± 0.318			
FATox (g·min^−1^) (n = 27 + 29)	Baseline	0.228 ± 0.168	0.270 ± 0.138	0.600	0.025	0.033
	Period I	0.231 ± 0.115	0.271 ± 0.181			
	Period II	0.259 ± 0.110†	0.269 ± 0.093			

*HR* heart rate, *VO*_*2*_ oxygen consumption, *RER* respiratory exchange ratio, *EE* energy expenditure, *CHOox* carbohydrate oxidation, *FATox* fat oxidation

*Statistically significant difference compared to baseline, *P* < 0.05

†Statistically significant difference compared to Period I, *P* < 0.05

**Table 5:** Blood-based markers of metabolic health at baseline and following the step-reduction (Period I) and exercise rehabilitation (Period II) periods. Data displayed as mean ± SD

		Intervention	Control	Time *P*-value	Group *P*-value	Time × Group interaction *P*-value
HCT (%)	Baseline	41.8 ± 2.3	41.6 ± 2.5	0.131	0.594	0.849
	Period I	41.5 ± 2.2	41.1 ± 2.9			
	Period II	41.6 ± 2.2	41.2 ± 2.6			
Gluc (mmol·L^−1^)	Baseline	5.47 ± 0.60	5.49 ± 0.56	0.035	0.845	0.606
	Period I	5.46 ± 0.72	5.44 ± 0.63			
	Period II	5.63 ± 0.78	5.54 ± 0.58			
HDL-C (mmol·L^−1^)	Baseline	1.62 ± 0.37	1.78 ± 0.54	0.006	0.187	0.065
	Period I	1.55 ± 0.36*	1.78 ± 0.54			
	Period II	1.66 ± 0.38†	1.81 ± 0.53			
LDL-C (mmol·L^−1^)	Baseline	2.88 ± 0.79	2.78 ± 0.91	< 0.001	0.666	0.536
	Period I	3.18 ± 0.99*	2.96 ± 1.03*			
	Period II	3.32 ± 0.92*	3.29 ± 1.12*†			
Trig (mmol·L^−1^)	Baseline	1.14 ± 0.44	1.19 ± 0.47	0.417	0.915	0.465
	Period I	1.23 ± 0.48	1.19 ± 0.52			
	Period II	1.27 ± 0.52	1.23 ± 0.67			
HOMA-IR (n = 28 + 31)	Baseline	1.64 ± 0.93	1.75 ± 1.04	0.777	0.997	0.768
	Period I	1.76 ± 1.01	1.95 ± 1.64			
	Period II	1.80 ± 1.18	1.95 ± 1.75			
Uric acid (µmol·L^−1^)	Baseline	337 ± 73	328 ± 81	< 0.001	0.689	0.140
	Period I	309 ± 64*	314 ± 71			
	Period II	311 ± 65*	300 ± 64*			

*HCT* hematocrit, *Gluc* glucose concentration, *HDL-C* high-density lipoprotein cholesterol concentration, *LDL-C* low-density lipoprotein cholesterol concentration, *Trig* triglyceride concentration, *HOMA-IR* homeostatic model assessment for insulin resistance

*Statistically significant difference compared to baseline, *P* < 0.05

†Statistically significant difference compared to Period I, *P* < 0.05

### Compliance influenced some of the intervention effects

From the secondary analyses, significant (3)Time × (3)Group interactions were observed for number of daily steps (F_(4, 116)_ = 58.32, *P* < 0.001), MVC (F_(4, 116)_ = 2.66, *P* = 0.036), basal HDL-C (F_(3.6, 103.2)_ = 3.81, *P* = 0.008), and for FATox (F_(4, 110)_ = 3.33, *P* = 0.013) when walking at 5 km·h^−1^.

**Fig. 3:**
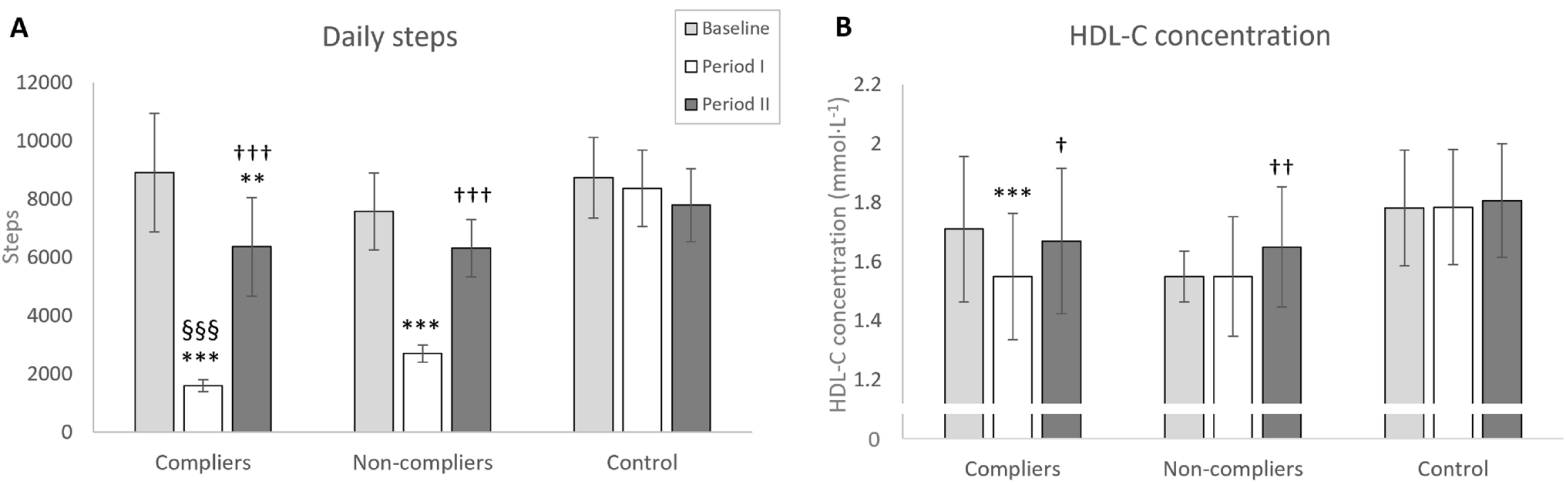
Mean ± 95% confidence intervals for daily steps (**A**) and high-density lipoprotein cholesterol concentration (**B**) in compliers, non-compliers, and non-training control groups. **P < 0.01, ***P < 0.001 compared to baseline. †*P* < 0.05, ††*P* < 0.01, †††*P* < 0.001 compared to Period I. §§§*P* < 0.001 compared to non-compliers

Both *compliers* and *non-compliers* reduced daily steps from baseline to Period I and then increased from Period I to Period II (Fig. 3A). However, the number of daily steps during Period I was significantly lower in compliers (1597 ± 316) than non-compliers (2697 ± 568, *P* < 0.001). There were no differences between groups during Period II.

Neither compliers nor non-compliers increased MVC from baseline to Period I, whereas the controls did (1864 ± 667–1959 ± 700 N, *P* = 0.041). Thereafter, the control group no longer showed any increases (*P* = 0.424) but both compliers (1850 ± 533–2036 ± 539 N, *P* = 0.016) and non-compliers (2018 ± 656–2338 ± 791 N, *P* < 0.001) increased over Period II.

Post hoc tests revealed that *compliers* reduced HDL-C concentration from baseline to Period I (*P* < 0.001) with a rebound to Period II (*P* = 0.021). *Non-compliers* did not reduce HDL-C concentration from baseline to Period I (*P* = 1.000) but showed increases from Period I to Period II (*P* = 0.008, Fig. 3B).

When walking at 5 km·h^−1^, *compliers* demonstrated an overall return toward fat oxidation from Period I to Period II (0.22 ± 0.10–0.29 ± 0.08 g·min^−1^, *P* = 0.023), despite the decrease from baseline (0.29 ± 0.10 g·min^−1^, *P* = 0.072) to Period I not being statistically significant. No changes were observed in *non-compliers* nor control.

### Adverse effects

No serious injuries were sustained due to the intervention. Drop-out was due to recurrence of dormant hip inflammation in one participant, one participant contracting a respiratory tract infection, and one participant slipping on ice injuring arm ligaments (intervention group), and one participant refusing follow-up testing due to a recurrence of previous back pain and one participant slipping on ice fracturing her clavicle (control group).

## Discussion

No significant changes occurred in the intervention group in leg lean mass, lower-limb force production capacity, increases in fat mass, nor altered blood glucose/insulin concentrations after step-reduction (Period I); thus, the hypothesis should be rejected. The only significant interaction was observed in bilateral leg press maximum voluntary contraction, where the intervention group increased force production after the exercise rehabilitation (Period II). Further, two weeks of step-reduction (~ 2242 steps, Δ = –68%) decreased HDL-C and increased LDL-C concentration (thus, negatively influencing HDL-C/LDL-C ratio). Four-week exercise rehabilitation returned HDL-C concentration to baseline levels.

### Body composition and physical function

Against the primary hypothesis, no loss in leg lean mass was observed in the present study as assessed by through DXA scanning. The method is valid (e.g., Cameron et al. 2020) and reliable (~ 1% CV%, Sillanpää et al. 2014; Walker and Häkkinen 2014) for this purpose, and it has identified lean mass/fat-free mass from step-reduction previously (Arentson-Lantz et al. 2019; Breen et al. 2013). However, the present study is not alone in observing maintained LLM after step-reduction (McGlory et al. 2018). One possibility for the unexpected maintenance of muscle tissue could be differing muscle signaling responses in older compared to young adults during muscle disuse. Suetta and colleagues (2012) showed heightened anabolic signaling responses (Akt and MGF) and blunted atrophy signaling responses (Atrogin-1 and MuRF-1) in their older adult group, which accompanied blunted muscle atrophy over a 2-week period of lower-limb immobilization. This should be further examined using the step-reduction model in young versus older adults to confirm.

In general, limited changes were observed in body composition in the present study. The only notable change was the significant ~ 0.5 kg (Δ2%) decrease in total fat mass in the intervention group following exercise rehabilitation (Period II). The initial months of exercise training has been shown to reduce fat mass (Eklund et al. 2016; Sillanpää et al. 2009), and a higher training frequency (i.e., number of sessions per week) has been suggested as being an important factor in fat loss (Eklund et al. 2016).

Although the 2-week step-reduction period did not lead to significant decreases in physical function as hypothesized, there was some evidence of interference in physical functioning in the present study. The control group improved maximum and initial (100 ms) lower-limb force production, lowered heart rate when walking at 3 and 5 km·h^−1^, as well as improved walking economy at 3 km·h^−1^ after Period I. Such improvements were not observed in the intervention group undergoing step-reduction. The intervention group only improved in these variables after the 4-week exercise rehabilitation (Period II) in addition to improved walking speed, 5 sit-to-stand performance, stair climb speed, and more favorable fat oxygenation during walking at 5 km·h^−1^.

Despite undergoing familiarization with the physical performance measures, it is not unusual for older adults to demonstrate statistically significant, but small-magnitude, improvements from repeated testing (Amarante do Nascimento et al. 2013). Several test sessions may be needed to establish stable baseline values. Further, maximum force production and functional capacity test performance have been observed to improve in non-training control groups (Holviala et al. 2006; Walker et al. 2017). Potentially, the frequent ~ 5–10% improvement in physical test performance of the control group during Period I could be explained by increased confidence to perform maximum testing, improved coordination, and initial neural adaptation, such as increased motor unit firing rate (Kamen and Knight 2004). This phenomenon of initial improvement when testing maximum performance may also partly explain why there was no decrease in MVC in the intervention group, despite step-reduction.

### Blood-based metabolic health

There were no alterations in basal blood glucose nor insulin concentrations, which meant that no change in HOMA-IR score was observed. Previous studies have repeatedly demonstrated negative effects of physical inactivity/muscle disuse on glucose regulation in both young and older adults (Breen et al. 2013; Krogh-Madsen et al. 2010; McGlory et al. 2018; Shad et al. 2019) and altered glucose regulation may even precede changes in body composition, observed after only 3 days of step-reduction (Knudsen et al. 2012). The studies that have observed altered glucose regulation have tended to employ oral glucose tolerance tests, which may be deemed a weakness of the present study, although one study with 2000 daily steps did not observe changes from an oral glucose tolerance test over a 7-day period (Arentson-Lantz et al. 2019). Such conflicting findings underscores the currently unresolved effect of short-term step-reduction on glucose regulation in healthy participants.

The most responsive secondary outcome to step-reduction and exercise rehabilitation was HDL-C concentration, which is usually considered to be important for maintaining good vascular health. Physical exercise has shown to increase HDL-C concentrations (Ihalainen et al. 2019). Using an apolipoprotein A_1_ knockout animal model to modify HDL levels, Lehti and colleagues (2013) showed negative effects on fasting glucose concentration, glucose tolerance test responses, and hepatic glycogen storage, which were not insulin dependent, as well as ex vivo skeletal muscle mitochondrial oxygen consumption and ATP synthesis in knockout mice. While maximal citrate synthase, a marker of mitochondrial content, was lowered after 7 days of step-reduction (Edwards et al. 2021), neither protein level nor phosphorylation level changes were observed in key markers of mitochondrial oxidative metabolism including AMPKα. The AMPK pathway has been proposed to mediate the effect of HDL on energy metabolism (Drew et al. 2009, 2011), and it may be that altered HDL is a precursor to subsequent alterations in energy metabolism and mitochondrial function.

Fasting glucose concentration and glucose regulation as measured by glucose tolerance test were compromised from 14 days of step-reduction < 1000 steps per day in prediabetic older adults, but no evidence of compromised mitochondrial content/function was observed (McGlory et al. 2018). Thus, it may be that a longer and/or more severe period of inactivity was needed to demonstrate dysfunction in glucose regulation and compromised metabolic health than implemented in the present study. No study to date has directly examined HDL particle concentration and mitochondrial content/function in relation to step-reduction or exercise rehabilitation; thus, this line of inquiry remains open. Nevertheless, regaining HDL-C concentration after exercise rehabilitation should be viewed as a positive finding to minimize mitochondrial damage (White et al. 2017). One potentially indirect link between HDL-C and mitochondrial function may have been observed in substrate utilization when walking at 5 km·h^−1^ since the exercise rehabilitation improved both HDL-C concentration and lowered RER, and these differences were exacerbated in both in *compliers* compared to *non-compliers*. Therefore, it is recommended to explore physical (in)activity effects on HDL and its influences on mitochondria in future.

It is beyond the present study to determine the mechanisms behind the changes in HDL-C and LDL-C concentrations of the intervention group. Nevertheless, one possibility for the initial decrease and then increased HDL-C concentration could be altered lecithin:cholesterol acyltransferase enzyme activity (Glomset 1979) influencing HDL-C formation. One interesting recent finding, that needs to be further explored, is that endurance running acutely reduced circulating levels of microRNA-33 (Faraldi et al. 2022). This has been implicated in the post-transcriptional regulation of ABCA-1 protein (Rye et al. 2014), which is a key element in regulating HDL-C concentration. While this proposal is speculative, it does provide a possible mechanism for the observed, rather fast-acting, response in HDL-C concentration to physical inactivity and exercise rehabilitation. The present study’s exercise-induced increases in HDL-C also agree with previous findings in older adults (Ihalainen et al. 2019).

Regarding altered LDL-C concentrations, it is unclear why both groups demonstrated significant increases throughout this study. Since the data collection period was October–December, it is possible that data collection coincided with the highest annual LDL-C concentrations (Sasaki et al. 1983; Wang et al. 2020). It is, therefore, possible that seasonal variation may have been a main factor influencing LDL-C findings, although other uncontrolled factors should not be discounted, e.g., diet.

Although uric acid concentrations were significantly altered in both groups during the present study, they remained well within normal limits (155–350 µmol·L^−1^ for females and 230–480 µmol·L^−1^ for males) and probably should be considered to be typical fluctuations rather than intervention effects.

### Daily steps

One interesting observation was that the number of daily steps did not return to baseline levels during the exercise rehabilitation period. The relative reduction of steps during the present study is well matched to those previously reported (~ 70% reduction) (Breen et al. 2013; McGlory et al. 2018), although some have shown even larger reductions (~ 82% in Devries et al. 2015). The only other study that tracked daily steps after the step-reduction period showed a return to baseline levels in older adults (McGlory et al. 2018). Some methodological differences do exist between the studies, which may explain the discord. McGlory et al. (2018) conducted their study in the spring whereas the present study was conducted in autumn/winter. In Jyväskylä, December 2021 was colder than average (–9.7 °C, i.e., 4.9 °C below historical average) with snow and particularly ice on the ground. Thus, the cold climate may have impacted habitual physical activity as there was a pattern for reduction (n.s.) also in the control group (~ 8732 to ~ 7790 steps), but this likely does not fully account for the observed reductions in daily steps during Period II in the intervention group. The present study employed an exercise rehabilitation program four times per week over a 4-week period, whereas McGlory et al. (2018) did not. Although it may be speculated that high-intensity resistance training may lead to reduced habitual physical activity, e.g., due to consequent fatigue, there appears no such evidence in the literature (Chin et al. 2006; Mosalman Haghighi et al. 2021). Nevertheless, the present study’s findings were likely not impacted by the ~ 17% lower number of daily steps during the exercise rehabilitation period as functional improvements were broadly observed following the 4-week exercise period and the mean number of steps (6328) was well above the proposed 4000 threshold for sedentary behavior-induced change in insulin sensitivity (Oikawa et al. 2019).

### Compliance and the potential influence of number of daily steps

It is unfortunate that over half (17/29) of the participants in the intervention group did not comply with the instructions to reduce steps below 2000. Rather than non-compliance per se, the higher step count than instructed was likely due to the participants being provided visual feedback by the pedometers (Silcott et al. 2011), which showed a mean bias of –611 steps in the present study. Nevertheless, in the present study the precise number of steps during the step-reduction period is meaningful as there were differences in the magnitude of the adaptation in HDL-C concentration and also in fat metabolism during walking. Although not completely resolved, 2000 daily steps are currently thought of as a threshold for inducing significant changes in physical function and muscle mass over short-term physical inactivity/muscle disuse (Oikawa et al. 2019). In support of this threshold, Arentson-Lantz and colleagues (2019) observed similar losses in maximum isokinetic torque and whole-body lean mass in participants undergoing bed rest with and without an intervention of 2000 daily steps.

Since most variables assessed in the present study showed no change during Period I, the 2000 daily step “threshold” does not appear a distinct “on/off” threshold. Nevertheless, some support for a 2000 daily step threshold was provided by oxidative phosphorylation substrate usage. Compliers in the present study showed evidence of disturbed fatty acid oxidation during walking at 5 km·h^−1^ after Period I, whereas non-compliers did not. Such metabolic inflexibility may be a sign of compromised metabolic health (Galgani et al. 2021). Further, only compliers demonstrated reduced HDL-C concentration during Period I, whereas neither non-compliers nor control did, although exercise rehabilitation robustly increased HDL-C concentration in both compliers and non-compliers.

### Study limitations

First, participants did not comply with the upper limit of daily steps (i.e., 2000) over the 2-week period in the intervention group, thus influencing statistical power. The literature overall appears to indicate that adverse changes in muscle mass and physical function occur over such short-term periods of physical inactivity when daily steps are limited to a maximum of 2000. Here, compliers’ daily steps were 1597 ± 316, while non-compliers’ were 2697 ± 568.

Second, diet was not controlled in the present study. Undertaking dietary analyses is a challenging task in older adults due to the need for manual (i.e., non-automatic) recording, potential of under-reporting and difficulties in recall, the demanding nature of tracking diet over a 6-week period, and indeed digitizing the data for a large group of individuals.

Third, an oral glucose tolerance test was not employed in the present study. Therefore, a more sensitive measure of glucose regulation is lacking and concluding that short-term physical inactivity does not lead to negative consequences for glucose regulation may be a Type II error.

## Conclusions

Two weeks of step-reduction did not decrease physical functioning, affect body composition (including the primary outcome measure: leg lean mass), nor alter most blood-based markers of metabolic health in well-functioning, asymptomatic older adults. Step-reduction did lead to decreased HDL-C concentration in those individuals that complied with the 2000 daily step instruction, but non-compliers showed no such decreases. This partly supports previous hypotheses regarding 2000 daily steps being a threshold for physiological dysfunction. Four weeks of exercise rehabilitation immediately after the step-reduction period increased HDL-C concentrations in both compliers and non-compliers. Further, the 4-week exercise rehabilitation, in itself, was successful in improving physical function and reducing fat mass, and older adults should be encouraged to engage in intensified physical activity following short-term periods of physical inactivity/muscle disuse.

## Data Availability

Data is available from the primary author upon reasonable request.
